# FLU, an amino acid substitution model for influenza proteins

**DOI:** 10.1186/1471-2148-10-99

**Published:** 2010-04-12

**Authors:** Cuong Cao Dang, Quang Si Le, Olivier Gascuel, Vinh Sy Le

**Affiliations:** 1 College of Technology, Vietnam National University Hanoi, 144 Xuan Thuy, Cau Giay, Hanoi, Vietnam; 2 Wellcome Trust Sanger Institute, Wellcome Trust Genome Campus, Hinxton, Cambridge, CB10 1SA, UK; 3 Methodes et Algorithmes pour la Bioinformatique, LIRMM, CNRS, Universite Montpellier II, Montpellier, France

## Abstract

**Background:**

The amino acid substitution model is the core component of many protein analysis systems such as sequence similarity search, sequence alignment, and phylogenetic inference. Although several general amino acid substitution models have been estimated from large and diverse protein databases, they remain inappropriate for analyzing specific species, e.g., viruses. Emerging epidemics of influenza viruses raise the need for comprehensive studies of these dangerous viruses. We propose an influenza-specific amino acid substitution model to enhance the understanding of the evolution of influenza viruses.

**Results:**

A maximum likelihood approach was applied to estimate an amino acid substitution model (FLU) from ~113, 000 influenza protein sequences, consisting of ~20 million residues. FLU outperforms 14 widely used models in constructing maximum likelihood phylogenetic trees for the majority of influenza protein alignments. On average, FLU gains ~42 log likelihood points with an alignment of 300 sites. Moreover, topologies of trees constructed using FLU and other models are frequently different. FLU does indeed have an impact on likelihood improvement as well as tree topologies. It was implemented in PhyML and can be downloaded from ftp://ftp.sanger.ac.uk/pub/1000genomes/lsq/FLU or included in PhyML 3.0 server at http://www.atgc-montpellier.fr/phyml/.

**Conclusions:**

FLU should be useful for any influenza protein analysis system which requires an accurate description of amino acid substitutions.

## Background

The majority of statistical methods used for analyzing protein sequences require an amino acid substitution model to describe the evolutionary process of protein sequences. Amino acid substitution models are frequently used to infer protein phylogenetic trees under maximum likelihood or Bayesian frameworks [[1,2], and references therein]. They are also used to estimate pairwise distances between protein sequences that subsequently serve as inputs for distance-based phylogenetic analyses [3]. Moreover, these models can be used for aligning protein sequences [4]. These and other applications of the amino acid substitution model are reviewed in [5].

Many methods have been proposed to estimate general amino acid substitution models from large and diverse databases [[1,6], and references therein]. These methods belong to either counting or maximum likelihood approaches. The first counting method was proposed by Dayhoff et al. [7] to estimate the PAM model. As more protein sequences accumulated, Jones et al. [8] used the same counting method to estimate the JTT model from a larger protein data set. However, the counting methods are limited to only closely related protein sequences.

The maximum likelihood method was proposed by Adachi and Hasegawa [9] to estimate the mtREV model from 20 complete vertebrate mtDNA-encoded protein sequences. The mtREV model outperformed other models when analyzing the phylogenetic relationships among species based on their mtDNA-encoded protein sequences. Whelan and Goldman [10] proposed a maximum likelihood method to estimate the WAG model from 182 globular protein families. The WAG model produced better likelihood trees than the Dayhoff and JTT models for a large number of globular protein families.

Recently, Le and Gascuel [6] improved the maximum likelihood method by incorporating the variability of evolutionary rates across sites into the estimation process. The method was used to estimate the so-called LG model from the Pfam database. Experiments showed that the LG model gave better results than other models both in terms of likelihood values and tree topologies.

Although a number of general models have been estimated from large and diverse databases comprising multiple genes and a wide range of species, they might be inappropriate for a particular set of species due to differences in the evolutionary processes of these species. A number of specific amino acid substitution models for important species have been introduced [11,12], e.g. HIV-specific models that showed a consistently superior fit compared with the best general models when analyzing HIV proteins.

In recent years, the world has encountered a series of emerging influenza epidemics, including H5N1 ('avian flu') or H1N1. These have caused serious problems in economics and human health. Theoretical and experimental studies have been extensively conducted to understand the evolution, transmission and infection processes of influenza viruses [13-17]. We propose here our FLU model which was specifically estimated for modeling the evolution of influenza viruses. Experiment results show that FLU is robust and better than other models in analyzing influenza proteins. Thus, it could enhance studies of the evolution of influenza viruses.

## Results and Discussion

We used the maximum likelihood approach introduced by Le and Gascuel [6] to estimate an influenza-specific amino acid substitution model (called FLU) from data set **D **comprising 992 influenza protein alignments. In the following sections, the main properties and performance of FLU in comparison with 14 widely used models will be analyzed.

### Model analysis

FLU, as an amino acid substitution model, includes a symmetric amino acid exchangeability matrix and an amino acid frequency vector. Thus, we analyze FLU with other models by comparing their amino acid exchangeabilities and frequencies. Table 1 presents low correlations between FLU and other models, which means that FLU is highly different from existing models. HIVb and HIVw are among the models that are most highly correlated with FLU, since they were also estimated from RNA virus proteins.

**Table 1:** The Pearson's correlations between FLU and 14 widely used models. The low correlations indicate that FLU is highly different from existing models.

model	exchangeability matrix	frequency vector
JTT	0.88	0.79
HIVb	0.86	0.71
HIVw	0.83	0.83
WAG	0.83	0.76
LG	0.82	0.71
CpREV	0.81	0.73
Blosum62	0.77	0.73
MtREV	0.77	0.48
RtREV	0.76	0.66
VT	0.75	0.76
MtMam	0.74	0.48
DCMut	0.74	0.69
Dayhoff	0.74	0.69
MtArt	0.70	0.45

**Figure 1:**
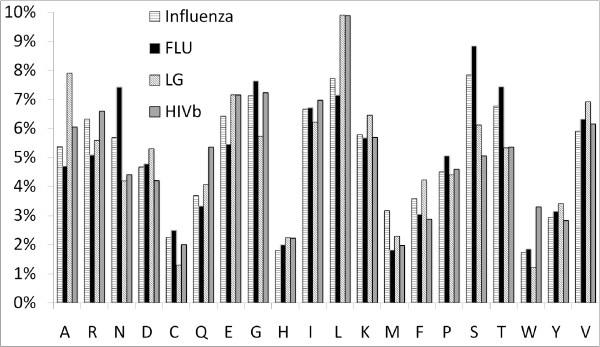
Amino acid frequencies of FLU, HIVb, LG models and the empirical frequencies counted from all alignments (denoted Influenza).

In the following, we compare FLU with HIVb (a HIV-specific model) and LG (the best general model) in detail. Figure 1 displays the amino acid frequencies of these models and the empirical amino acid frequencies (denoted Influenza) that were counted from all alignments of data set **D**. Amino acid frequencies of FLU and Influenza are nearly identical (correlation ~0.94), the correlation being much higher than that of Influenza with the 2 other models, HIVb (~0.84) and LG (~0.84). Notably, we observe large differences between the amino acid frequencies of Influenza and the others. For example, the frequency of leucine (L) in Influenza (~7%) is much lower than that in HIVb (~10%) and LG (~10%). These results indicate that FLU represents the amino acid frequencies of influenza proteins more accurately than other models.

**Figure 2:**
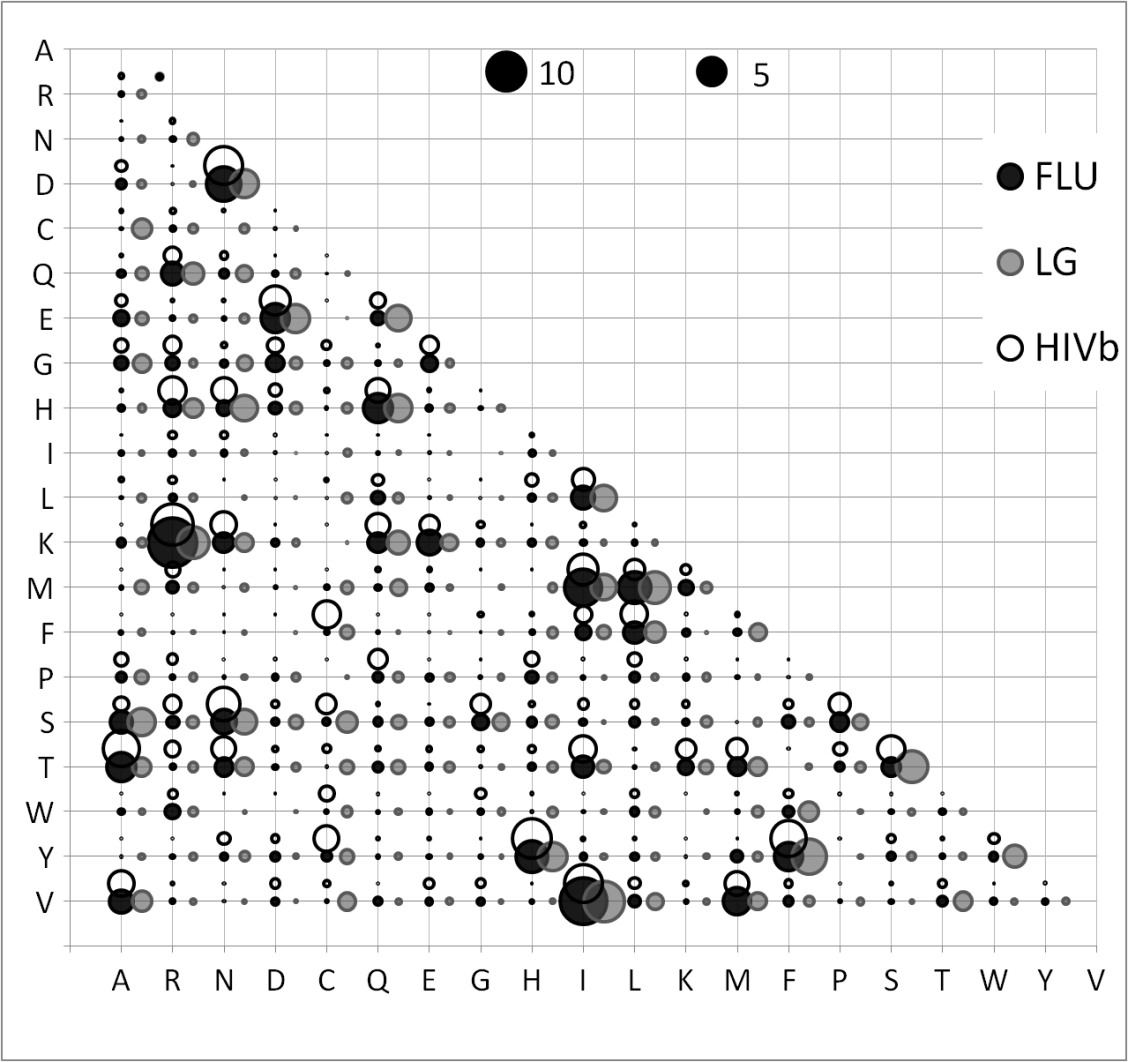
**The exchangeability coefficients in FLU, HIVb and LG models**. The black bubble at the intersection of line *X *and column *Y *presents the exchangeability between amino acid *X *and amino acid *Y *in FLU. Similarly, the grey and white bubbles present exchangeabilities between amino acids in the LG and HIVb models, respectively. These bubbles show remarkable differences between these models.

**Figure 3:**
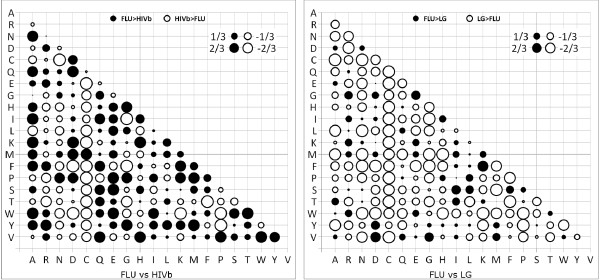
**The bubbles display the relative differences between exchangeability coefficients in FLU and HIVb (left), and FLU with LG (right)**. On the left side, each bubble represents the value of (*FLU*_*ij *_- *HIVb*_*ij*_)/(*FLU*_*ij *_+ *HIVb*_*ij*_) where *FLU*_*ij *_(*HIVb*_*ij*_) is the exchangeability coefficient in FLU (HIVb). Values 1/3 and 2/3 mean that the FLU coefficient is 2 and 5 times as large as that of HIVb, respectively. Values -1/3 and -2/3 mean that HIVb is 2 and 5 times larger than FLU, respectively. Similar explanations can be also given on the right side, but now between FLU and LG models.

The exchangeability coefficients of FLU, HIVb, and LG models (Figure 2), in principle, describe similar biological, chemical and physical properties of the amino acids, e.g. the high exchange rate between lysine (a positively charged, polar amino acid) and arginine (a positively charged, polar amino acid) or the low exchange rate between lysine and cysteine (a neutral, nonpolar amino acid). However, they differ considerably when we look in their relative differences (Figure 3). For example, 41 out of 190 coefficients in FLU are at least 5 times as large as corresponding ones in the HIVb model. Table 2 summarizes the relative differences between FLU and HIVb, LG models.

**Table 2:** Relative differences between FLU and HIVb (LG) models.

	FLU > HIVb	HIVb > FLU	FLU > LG	LG > FLU
Twice	67	40	20	90
Five	41	21	2	53

The value at the row 'Twice' and column 'FLU>HIVb' indicates the number of exchangeability coefficients in FLU that are at least twice as large as corresponding ones in the HIVb model. Similar explanations can be given for other entries.

In a nutshell, FLU is very different from existing models in both amino acid exchangeabilities and frequencies.

### FLU performance

We compared the performance of FLU and other models in constructing maximum likelihood trees for influenza protein alignments. Maximum likelihood trees were constructed by PhyML with 4 discrete gamma rate categories (+Γ = 4), invariant sites (+I), and -F/+F options [18].

#### Global test

In the global test, we used FLU and other models to construct maximum likelihood trees for 992 protein alignments of **D**. Since we estimated and tested FLU on the same data set **D**, it contains more free parameters than other models, i.e. 208 with -F option or 189 with +F option. To compare the performance of FLU and other models, the AIC criterion was used [19].

The average AIC of FLU is higher than that of other models (Table 3). For example, FLU gains 0.3 AIC per site when compared with the second best model, HIVb. In the case where 2 models have the same number of free parameters, 0.3 AIC per site is equivalent to ~45 log likelihood points per alignment of 300 sites. The last column of Table 3 shows the AIC differences between +F and -F options. The +F option would improve the AIC only when the amino acid frequencies of the model are significantly different from the empirical frequencies. However, the +F option might lead to the loss of AIC due to the penalty of 19 additional free parameters. Table 3 shows that the +F option did not improve the AIC for most of the models due to the slight difference between the Influenza and the amino acid frequencies of the models, except MtREV, MtMam, and MtArt estimated from mitochondrial proteins. In these cases, the +F option significantly improved the AIC because of the high difference between the amino acid frequencies of influenza and mitochondrial proteins (correlation ~0.54).

**Table 3:** Average AIC per site of FLU and other models. FLU has better AIC than other models.

	without F option	with F option	difference between
	(-F)	(+F)	+F and -F options
FLU	-21.01	-21.09	-0.08
HIVb	-21.31	-21.34	-0.03
JTT	-21.37	-21.37	-0.00
HIVw	-21.43	-21.42	0.01
CpREV	-21.49	-21.54	-0.05
LG	-21.57	-21.56	0.01
WAG	-21.58	-21.51	0.07
VT	-21.79	-21.68	0.11
Dayhoff	-21.79	-21.62	0.17
DCMut	-21.79	-21.62	0.17
RtREV	-21.80	-21.70	0.10
Blosum62	-21.85	-21.82	0.03
MtREV	-22.48	-21.76	0.72
MtMam	-22.73	-21.97	0.76
MtArt	-22.86	-22.15	0.71

#### Two-fold cross validation

In the two-fold cross validation, we randomly divided **D **into halves **D**_1 _and **D**_2 _where either one served as the learning data set and the other acted as the testing data set. Due to the low number of protein types (see Table 4), **D**_1 _and **D**_2 _might contain alignments of the same protein types. We first estimated FLU_1 _(FLU_2_) model from **D**_1 _(**D**_2_), and then used FLU_1 _(FLU_2_) to construct maximum likelihood trees for alignments of **D**_2 _(**D**_1_). Consequently, we obtained 992 maximum likelihood trees inferred using either FLU_1 _or FLU_2_. For the sake of simplicity, we denote FLU as the overall model for FLU_1 _and FLU_2 _in analyzing the two-fold cross validation. Since learning and testing data sets are independent, there is no penalty for additional free parameters when comparing FLU with other models, i.e., we could directly compare log likelihoods of trees inferred using FLU and other models.

**Table 4:** A summary of influenza viruses.

	Type A	Type B	Type C	proportion (%)
HA	∨	∨	∨	30,63
NA	∨	∨		14,67
PA	∨	∨		9,06
PB2	∨	∨	∨	8,93
PB1	∨	∨	∨	7,97
NS1	∨	∨	∨	7,65
NP	∨	∨	∨	6,87
M2	∨			4,13
NS2	∨	∨	∨	3,49
PB1-F2	∨			3,29
M1	∨	∨	∨	3,10
NB		∨		0,11
BM2		∨		0,04
CM2			∨	0,03
P3			∨	0,02

The last column shows proportions of proteins used to estimate the FLU model.

It is clear from Tables 5 and 6 that FLU outperforms all other models. It helps to construct the best likelihood trees for 680 out of 992 alignments (69%) and the second best trees for 131 other alignments (13%). FLU trees also have the highest average likelihoods, which is 0.14 log likelihood point per site higher than the second best model, HIVb (Table 7). This means that FLU gains about ~42 log likelihood points on average when applied to an alignment of 300 amino acids. HIV models, as expected, are the second and third best models since they were also estimated from RNA virus proteins. Since HA and NA proteins are the most crucial proteins of influenza viruses, a large number of HA and NA protein sequences were available to estimate the model (see Table 4). FLU outperforms other models in ~98% of HA and NA alignments. It is significantly better than HIVb in ~95% (~92%) of HA (NA) alignments. However, it is worse than HIVb when analyzing M2 and PB1-F2 protein alignments.

**Table 5:** Comparisons of FLU and 14 other models in constructing maximum likelihood trees (-F option).

	1^*st*^	2^*nd*^	3^*rd*^	4^*th*^	5^*th*^	6^*th*^	7^*th*^	8^*th*^	9^*th*^	10^*th*^	11^*th*^	12^*th*^	13^*th*^	14^*th*^	15^*th*^
FLU	680	129	147	19	2	4	4	4	1	1	1	0	0	0	0
HIVb	200	405	198	46	33	64	18	8	7	6	7	0	0	0	0
HIVw	91	115	200	178	64	58	144	20	29	16	16	61	0	0	0
JTT	14	274	290	398	14	0	1	0	1	0	0	0	0	0	0
LG	5	15	26	75	168	394	189	15	64	21	16	4	0	0	0
CpREV	2	25	54	204	542	112	13	20	8	7	4	1	0	0	0
WAG	1	28	70	55	134	278	357	43	25	1	0	0	0	0	0
Dayhoff	0	1	0	1	8	18	94	196	209	235	200	24	5	1	0
VT	0	0	3	9	17	30	74	226	192	164	178	71	24	4	0
Blosum62	0	0	3	7	8	18	28	103	84	139	95	436	24	47	0
DCMut	0	0	1	0	1	9	35	103	176	207	249	199	8	4	0
RtREV	0	0	0	0	1	5	29	234	175	174	190	157	14	13	0
MtMam	0	0	0	0	0	2	5	12	10	15	16	14	49	638	230
MtREV	0	0	0	0	0	0	1	8	11	6	20	25	849	69	3
MtArt	0	0	0	0	0	0	0	0	0	0	0	0	19	216	757

The number on the cell of model M and column *p *indicates the number of alignments where M model stands at the rank *p *over 15 models tested. For example, FLU model stands at the first rank for 680 out of 992 alignments.

**Table 6:** Comparisons of FLU and 14 other models in constructing maximum likelihood trees (+F option).

	1^*st*^	2^*nd*^	3^*rd*^	4^*th*^	5^*th*^	6^*th*^	7^*th*^	8^*th*^	9^*th*^	10^*th*^	11^*th*^	12^*th*^	13^*th*^	14^*th*^	15^*th*^
FLU	635	123	202	19	5	2	2	1	2	0	1	0	0	0	0
HIVb	196	375	105	109	61	25	21	22	22	16	35	5	0	0	0
HIVw	148	146	290	73	36	41	22	11	17	56	36	93	19	3	1
JTT	6	168	218	540	23	20	9	5	1	2	0	0	0	0	0
MtREV	3	1	2	9	77	127	102	66	43	38	115	91	307	10	1
MtMam	2	4	6	7	52	62	53	60	42	62	39	92	71	343	97
WAG	1	166	124	52	33	96	146	130	89	63	55	25	12	0	0
CpREV	1	3	5	18	451	159	158	64	95	28	4	3	3	0	0
VT	0	3	11	21	34	35	46	80	83	135	73	101	206	151	13
LG	0	1	16	110	131	240	134	83	140	53	42	40	2	0	0
Dayhoff	0	1	11	19	60	93	151	227	145	147	91	28	13	5	1
Blosum62	0	1	1	3	1	2	5	11	25	20	115	192	106	203	307
DCMut	0	0	1	11	24	73	108	153	213	143	145	88	26	6	1
MtArt	0	0	0	1	3	1	5	4	4	5	12	32	137	219	569
RtREV	0	0	0	0	1	16	30	75	71	224	229	202	90	52	2

The number on the cell of model M and column *p *indicates the number of alignments where M model stands at the rank *p *over 15 models tested. For example, FLU model stands at the first rank for 635 out of 992 alignments.

**Table 7:** Comparisons of FLU and 14 other models in constructing maximum likelihood trees.

	LogLK/site	LogLK/site
	without F option (-F)	with F option (+F)
FLU	-10.51	-10.49
HIVb	-10.65	-10.61
HIVw	-10.71	-10.65
JTT	-10.68	-10.63
LG	-10.78	-10.82
cpREV	-10.74	-10.93
WAG	-10.78	-10.70
Dayhoff	-10.89	-10.71
VT	-10.89	-10.78
Blosum62	-10.92	-10.72
DCMut	-10.89	-10.75
RtREV	-10.89	-10.85
MtMam	-11.36	-10.75
MtREV	-11.23	-11.01
MtArt	-11.42	-10.79

FLU trees have the highest average likelihoods.

The likelihood difference between 2 trees inferred using 2 different models *M*_1 _and *M*_2 _might fluctuate due to various error factors, e.g., numerical problems and local optimizations. To assess the statistical significance of the difference between *M*_1 _and *M*_2_, we used a simple nonparametric version of the Kishino-Hasegawa (KH) test [20] as used in [6]. As explained in [6], the test avoids any normality assumption and selection bias that would favor one model compared with the other (refer to [6,21] for detailed explanations and calculations). Table 8 shows that FLU is significantly better than other models for the majority of alignments. For example, the KH test determined 484 (~49%) alignments where FLU trees had significantly higher likelihood values than HIVb trees. The number increases to 731 (~74%) or 907 (~92%) when compared with the JTT and LG, respectively. FLU was significantly worse than one of 14 compared models in only ~7% of alignments. These comparisons lead to the conclusion that FLU describes the evolution of influenza viruses better than other models, thus resulting in more accurate phylogenetic trees.

**Table 8:** Pairwise comparisons between FLU and HIVb, HIVw, JTT, LG models.

		LogLK/site		#*M*_1 _> *M*_2_	#*M*_2 _> *M*_1_
*M*_1_	*M*_2_		*M*_1 _> *M*_2_	(*p *< .05)	(*p *< .05)
FLU (-F)	HIVb (-F)	0.14	696	484	49
FLU (-F)	HIVw (-F)	0.19	843	689	46
FLU (-F)	JTT (-F)	0.17	926	731	10
FLU (-F)	LG (-F)	0.26	971	907	6
FLU (+F)	HIVb (+F)	0.12	674	437	89
FLU (+F)	HIVw (+F)	0.16	734	561	84
FLU (+F)	JTT (+F)	0.13	958	755	3
FLU (+F)	LG (+F)	0.23	988	954	0

LogLK/site: the log likelihood difference between trees inferred using *M*_1 _and *M*_2_; a positive (negative) value means *M*_1 _is better (worse) than *M*_2_. #*M*_1 _> *M*_2_: the number of alignments among 992 alignments where *M*_1 _results in better likelihood value than *M*_2_. #*M*_1 _> *M*_2 _(*p *< 0.05): the number of alignments where the Kishino-Hasegawa test indicates that M_1 _is significantly better than *M*_2_. #*M*_2 _> *M*_1 _(p < 0.05): the same as #*M*_1 _> *M*_2_, but now *M*_2 _is significantly better than *M*_1_.

### Tree analysis

We observed a large number of alignments where tree topologies of FLU and other models were different (Table 9). For example, FLU trees and HIVb trees are topologically different for 917 (~92%) alignments, of which FLU is better than the HIVb for 655 (~72%) alignments.

**Table 9:** Pairwise comparisons between FLU and HIVb, HIVw, JTT, LG models.

			#*T*_1 _> *T*_2_	#*T*_2 _> *T*_1_
*M*_1_	*M*_2_	#*T*_1 _> *T*_2_	(*p *< .05)	(*p *< .05)
FLU (-F)	HIVb (-F)	655/917	454	40
FLU (-F)	HIVw (-F)	792/932	655	41
FLU (-F)	JTT (-F)	890/938	710	6
FLU (-F)	LG (-F)	921/935	868	5
FLU (+F)	HIVb (+F)	627/916	412	83
FLU (+F)	HIVw (+F)	701/932	540	78
FLU (+F)	JTT (+F)	887/912	705	3
FLU (+F)	LG (+F)	922/924	897	0

*T*_1 _(*T*_2_) is the tree inferred using *M*_1 _(*M*_2_) model. #*T*_1 _> *T*_2_: the number of alignments where topologies of *T*_1 _and *T*_2 _are different and the likelihood of *T*_1 _is higher than the likelihood of *T*_2 _(the first number), and the number of alignments where topologies of *T*_1 _and *T*_2 _are different (the second number). #*T*_1 _> *T*_2 _(*p *< 0.05): special cases of #*T*_1 _>*T*_2_, where *T*_1 _is significantly better than *T*_2_. #*T*_2 _> *T*_1 _(*p *< 0.05): the same as #*T*_1 _> *T*_2 _(*p *< 0.5), but now *T*_2 _is significantly better than *T*_1_.

To measure the difference between 2 tree topologies, we used the Robinson-Fould (RF) distance, which is the number of bi-partitions present in one of the two trees but not the other, divided by the number of possible bi-partitions. Thus, the smaller the RF distance between 2 trees, the closer their topologies. Note that the RF ranges from 0.0 to 1.0.

**Figure 4:**
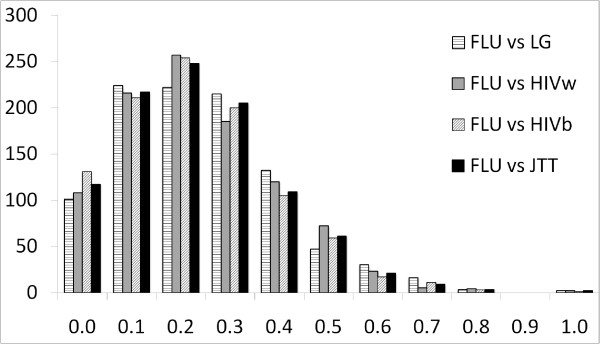
**The Robinson-Foulds distance between trees inferred using FLU and HIVb (LG, JTT, HIVw) models**. The horizontal axis indicates the RF distance between 2 tree topologies, whereas the vertical axis indicates the number of alignments.

Figure 4 shows that tree topologies inferred using FLU are highly different from those inferred using other models. For example, the RF distance between FLU trees and HIVb trees is ~0.2 (~0.4) for about 25% (12.5%) of alignments. The average branch length of FLU trees (0.037) is longer than that of trees inferred using general trees, e.g. LG (0.032), JTT (0.031). This finding indicates that FLU trees capture more hidden substitutions that might have occurred along the branches and therefore might better characterize the evolutionary patterns of influenza viruses than trees inferred using general models (see [22] for discussions on tree length).

### Robustness of model

We investigated the robustness of FLU by measuring the correlations between FLU, FLU_1 _and FLU_2_. Table 10 shows extremely high correlations (> 99%) between FLU, FLU_1 _and FLU_2 _in both amino acid frequencies and exchangeability coefficients. Thus, the data set **D **is sufficiently large to estimate a robust amino acid substitution model for influenza proteins.

**Table 10:** Correlations between FLU, FLU_1 _and FLU_2 _models.

	exchangeability matrix	frequency vector
FLU vs FLU_1_	99.95%	99.98%
FLU vs FLU_2_	99.95%	99.98%
FLU_1_vs FLU_2_	99.81%	99.94%

The exchangeability (frequency) column gives the correlations between exchangeability matrices (frequency vectors) of these models.

We also examined the influence of the temporal aspect of influenza evolution on FLU. To this end, the data set **D **was divided into 2 nearly equal subsets **D**_*t*1 _(27,752 protein sequences before 2004) and **D**_*t*2 _(23,397 protein sequences since 2004). We used subset **D**_*t*1 _(**D**_*t*2_) to estimate model FLU_*t*1 _(FLU_*t*2_). FLU_*t*1 _and FLU_*t*2 _were nearly identical (correlation ~0.99). Moreover, FLU_*t*1 _and FLU_*t*2 _were highly correlated to FLU (correlation ~0.97). The high correlations indicate that the influence of the temporal aspect of influenza evolution on estimating the amino acid substitution model is insignificant. Thus, FLU is applicable to analyze both old and recent influenza proteins.

## Conclusions

We propose the FLU model that has been specifically estimated for modeling the evolution of influenza viruses. Analyses revealed significant differences between FLU and existing models in both amino acid frequencies and exchangeability coefficients. Experiments showed that FLU better characterizes the evolutionary patterns of influenza viruses than general models.

Both the global test and 2-fold cross validation confirmed that FLU is better than existing models in constructing maximum likelihood trees. Using the KH test, FLU proved significantly better than other models for a majority of alignments tested. Nevertheless, there were a few alignments (typically from M2 and PB1-F2 proteins) where FLU was significantly worse than the HIV-specific models or general models, e.g. LG, or JTT. In this study, amino acid sequences were aligned by Muscle [23] to produce alignments that serve as inputs for estimating FLU. Recently, Liu et al. [24] proposed a method for coestimating sequence alignments and phylogenetic trees, and showed that it improved tree and alignment accuracy compared with 2-phase methods for large DNA data sets. Although previous studies showed that models estimated using near-optimal phylogenetic trees are relatively stable [[10], and references therein], it would be interesting to assess the influence of the coestimation method on the estimation of amino acid substitution models in future work. The occurrence of homologous recombination within influenza virus genes has been reported, however, it is rare and controversial [25,26]. Therefore, the FLU was estimated in a standard phylogenetic framework. The effect of the homologous recombination, if it occurs at all, on the FLU model would be discovered in future work. In summary, FLU model is useful for any influenza protein analysis system that demands an accurate description of amino acid substitutions. It should enhance our understanding of the evolution, transmission and infection processes of influenza viruses.

## Methods

### Data

Influenza viruses are RNA viruses from the Orthomyxoviridae family, which is divided into 3 types: influenzas A, B, and C. Influenza A viruses frequently cause serious epidemics and pandemics, such as Spanish flu H1N1, Asian flu H2N2, Hong Kong flu H3N2, or avian flu H5N1 (see Table 4 for a short summary of influenza viruses). Influenza viruses have been isolated since the beginning of the 20th century, and a huge number of their proteins have been sequenced and stored at the NCBI [13,16].

To estimate the amino acid substitution model for influenza viruses, we downloaded the entire influenza database at NCBI (July 26th 2009 version) [16], including 112,450 protein sequences (103,626 for A; 7,892 for B; and 932 for C). The sequences were processed before estimating the model.

• *Cleaning step*: Only distinct sequences were kept. The set consisted of 51,061 sequences, i.e. 46,909 for A; 3,845 for B; and 307 for C.

• *Dividing step*: These distinct sequences were randomly divided into small groups such that each group contained from 5 to 100 homologous sequences (the same protein type) of the same virus type. This resulted in 1046 groups.

• *Aligning step*: The 1046 groups were aligned by Muscle, a multiple alignment program [23]. The alignments were cleaned by the GBLOCKS [27] to eliminate sites containing many gaps. We selected 992 alignments which contain at least 5 sequences and 50 sites for estimating the model.

### Model

We assume, as usual, that amino acid sites evolve independently, and the process has remained constant throughout the course of evolution. The substitution process between amino acids is modeled by a time-homogeneous, time-continuous, time-reversible, and stationary Markov process [[1,2,28], and references therein]. The central component of the process is the so-called instantaneous substitution rate 20 × 20-matrix **Q **= {*q*_*xy*_} where *q*_*xy *_(*x *≠ *y*) is the number of substitutions from amino acid *x *to amino acid *y *per time unit. The diagonal elements *q*_*xx *_are assigned such that the sum of each row equals zero. The matrix **Q **can be decomposed into symmetric exchangeability rate matrix *R *= {*r*_*xy*_} and amino acid frequency vector *π *= {*π*_*x*_} such that *q*_*xy *_= *r*_*xy*_*π*_*y *_and *q*_*xx *_= -Σ_*y*≠*x *_*q*_*xy*_.

The likelihood of a multiple sequence alignment *D *= {*d*_1_, ..., *d*_*n*_} of *n *sites given their phylogenetic tree *T *and the model **Q **is(1)

where *L*(*T*, **Q**|*d*_*i*_) is the likelihood of site *d*_*i *_given tree *T *and model **Q **that can be efficiently calculated by a pruning algorithm [29].

In Equation 1, we assumed the same substitution rate across amino acid sites. To incorporate the variability of substitution rates across sites we used the combination of invariant model [30,31] and Γ-distribution model [32]. The heterogeneous rate model **r **assumes a fraction *θ*_inv _of sequence sites to be invariant, and other sites are variant with global substitution rates following the Γ-distribution [33].

The likelihood of *D *given the phylogenetic tree *T*, substitution model **Q**, and rate model **r **is computed as

where *L*(*inv*|*d*_*i*_) is the likelihood of site *d*_*i *_following the invariant model, that is, *L*(*inv*|*d*_*i*_) is equal to *π*_*x *_if site *d*_*i *_is constant and contains only amino acid *x*, otherwise zero when the site *d*_*i *_is not constant; *r*_*c*_*T *denotes the tree *T *with all branch lengths being multiplied by *r*_*c*_.

### Model estimation

Given a set of *m *protein alignments **D **= {*D*_1_, ..., *D*_*m*_}, the substitution model **Q **can be estimated by the counting or the maximum likelihood approach [[1], and references therein]. A number of studies have shown that the maximum likelihood approach can avoid systematic errors and makes more efficient use of information in the protein alignments compared with the counting approach [10]. We applied the maximum likelihood approach, introduced by Le and Gascuel in [6], to estimate the model **Q**.

**Figure 5:**
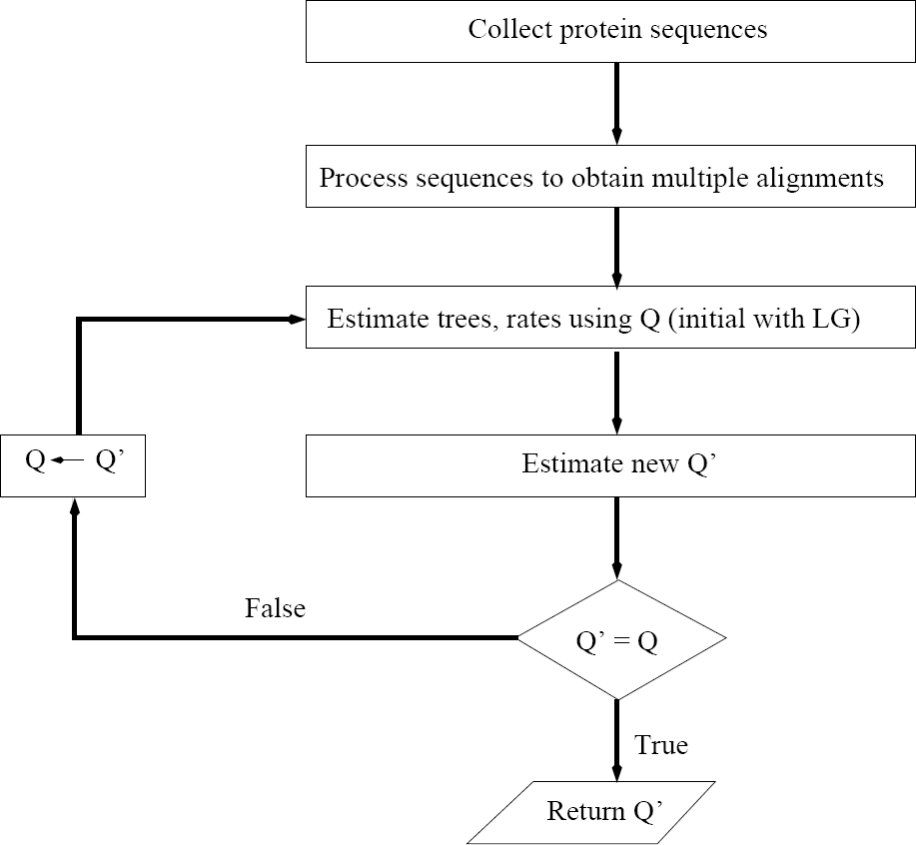
**Flowchart to estimate the influenza-specific amino acid substitution model**.

The model **Q **is estimated by maximizing the likelihood *L*(**D**):(2)

where *T*_*i *_and **r**_*i *_are the phylogenetic tree and rate model of the alignment *D*_*i*_, respectively. Optimizing the likelihood *L*(**D**) is a difficult problem because we have to construct all phylogenetic trees (topologies and branch lengths), **Q **coefficients and rate parameters. Fortunately, previous studies discovered that the estimated coefficients of **Q **remained nearly unchanged when near-optimal phylogenetic trees and rate parameters were used [[10], and references therein]. Thus, the Equation 2 can be simplified and approximated to:(3)

where  and  are near-optimal phylogenetic tree and rate model of *D*_*i*_, respectively. We designed a 5-step procedure to estimate the model **Q **(see Figure 5):

• *Step 1*: Collect all influenza protein sequences from the influenza database at NCBI (112,450 protein sequences).

• *Step 2*: Process retrieved sequences as described in the 'Data' section to obtain 992 multiple alignments.

• *Step 3(***Q **= *LG as the default)*: Estimate trees, rates, etc., using **Q **and the phylogenetic software PhyML [18].

• *Step 4: *Estimate a new model **Q**' using the approach introduced in [6] and the XRate software [34].

• *Step 5: *Compare 2 models **Q **and **Q**'. If **Q**' is nearly identical to **Q**, return **Q**' and consider it as the model for influenza viruses. Otherwise, **Q ← Q**' and goto *Step 3*.

FLU was obtained after two iterations.

## Authors' contributions

CCD, QSL, VSL, and OG discussed ideas. CCD implemented programs, conducted experiments, and wrote the draft manuscript. QSL and VSL designed experiments and revised the manuscript. All authors read and approved the final manuscript.
